# Urban Habitat Mosaics Structure Gene Flow but Preserve Genetic Diversity in an Invasive Termite

**DOI:** 10.1002/ece3.74439

**Published:** 2026-10-19

**Authors:** Emilie Gheyle, Edouard Duquesne, Yves Roisin, Denis Fournier

**Affiliations:** ^1^ Evolutionary Biology & Ecology Université libre de Bruxelles (ULB) Brussels Belgium

**Keywords:** biological invasions, Caribbean, colony breeding system, gene flow, genetic diversity, *Heterotermes tenuis*, landscape genetics, microsatellites, termites, urbanization

## Abstract

Urbanization is expanding rapidly in tropical regions, yet its effects on the genetic structure and connectivity of invasive social insects remain poorly understood. Here, we investigated how urban and forest habitat contexts are associated with genetic diversity, population structure, and colony organization in the invasive termite *Heterotermes tenuis* (Hagen, 1858) across three paired urban‐forest sites in Guadeloupe, using 18 newly developed polymorphic microsatellite loci. A total of 414 workers from 50 nests were genotyped. Across habitats, we found no differences in allelic richness, observed heterozygosity, expected heterozygosity, or inbreeding coefficients, suggesting no generalized loss of neutral genetic diversity in urban populations. In contrast, analyses of population structure revealed significant genetic differentiation between urban and forest habitats within each site pair, with habitat explaining 14%–22% of the genetic variation. Isolation by distance was detected in only one site, and a landscape‐level MLPE model indicated that habitat context was a stronger predictor of genetic differentiation than geographic distance. Colony genetic structure was consistent across habitats: no mixed‐family nests were detected, and extended family colonies predominated in both urban and forest environments. Together, these results indicate that urban and forest populations maintain comparable neutral genetic diversity while exhibiting moderate habitat‐associated genetic structuring. However, because habitat type covaried with several environmental characteristics and historical introduction structure cannot be excluded, this differentiation cannot be attributed to urbanization alone. These findings highlight the importance of considering habitat‐associated genetic structure and colony organization when assessing the spread and management of invasive termites in tropical urban landscapes.

## Graphical abstract

We investigated how urbanization shapes genetic diversity, population structure, and colony organization in the invasive termite *Heterotermes tenuis* across paired urban‐forest sites in Guadeloupe, using 18 microsatellite loci to genotype 414 workers from 50 nests. While neutral genetic diversity remained comparable between habitats, significant genetic differentiation was detected between urban and forest environments, with habitat type explaining 14%–22% of total genetic variation and acting as a stronger driver of differentiation than geographic distance. These results indicate that the urban matrix promotes moderate habitat‐associated genetic structuring without acting as an absolute barrier to gene flow, underscoring the value of integrating invasive termites into urban landscape genetics and biosecurity monitoring in tropical regions.

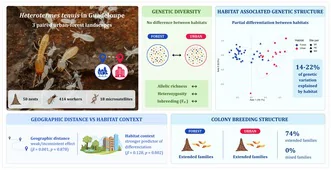

## 1 Introduction

Habitat loss and fragmentation are among the most prevalent consequences of global environmental change and represent major threats to biodiversity at local, regional, and global scales (IPBES 2019; Pereira et al. 2010; Hanski 2011; Pachauri et al. 2014; Newbold et al. 2015). Urbanization is a particularly rapid and widespread form of habitat transformation, converting natural landscape into built environments dominated by impervious surfaces and infrastructure such as buildings and roads (Arnold Jr. and Gibbons 1996; McKinney 2002; Santos 2020). Since the early 1990s, urban areas have more than doubled in extent (IPBES 2019; Ren et al. 2023), and nearly 70% of the world's population is projected to live in cities by 2050 (United Nations 2019, 2022). Urban expansion drives habitat fragmentation and degradation, alters microclimatic conditions through soil structure and chemistry (Pouyat et al. 1995; Biasioli et al. 2006; Liu et al. 2019), and increases multiple forms of pollution with major consequences for biodiversity and ecosystem functioning (Biasioli et al. 2006; Stone 2017; Huang et al. 2019; Chen et al. 2020). Understanding how these transformations affect ecological and evolutionary processes is therefore a major challenge for contemporary biodiversity research. Although the effects of urbanization on termite communities, functional diversity, and morphology have received increasing attention (Eggleton et al. 1996; Liu et al. 2019; Duran‐Bautista et al. 2020, 2024; Santos 2020; Duquesne et al. 2026), their consequences for genetic diversity, population connectivity, and colony breeding systems remain comparatively understudied.

Urbanization can influence population genetics through multiple, potentially opposing mechanisms. Habitat fragmentation may reduce effective population size, restrict dispersal, and increase genetic drift, ultimately leading to reduced genetic diversity and increased differentiation among populations (Hanski 2005, 2011; Jaquiéry et al. 2009; Lowe and Allendorf 2010; Baguette et al. 2013; Fletcher et al. 2016; Fusco et al. 2021). At the same time, urban landscapes may create novel dispersal pathways through transportation networks and human‐mediated movement, facilitating connectivity among otherwise isolated populations (McKinney 2008; Buczkowski and Richmond 2012; Bellard et al. 2016; Miles et al. 2018; Urban et al. 2024). Consequently, urban populations may exhibit either reduced genetic diversity and increased isolation, or maintained diversity despite substantial genetic structuring. Distinguishing between these alternative outcomes is central to understanding how urbanization shapes contemporary evolutionary dynamics.

Despite growing interest in urban landscape genetics, current knowledge remains taxonomically and geographically biased. Most studies have focused on plants, birds, and mammals from temperate regions, particularly Europe and North America, whereas terrestrial arthropods from tropical ecosystems remain comparatively understudied (Fenoglio et al. 2020; Collins et al. 2021; Fusco et al. 2021; Rega‐Brodsky et al. 2022). Expanding research across a broader range of taxa and biogeographic contexts is therefore essential to identify general and taxon‐specific responses to urbanization (Fusco et al. 2021).

Among arthropods, termites provide a particularly relevant but overlooked model system. Together with ants, termites account for nearly half of global insect biomass while representing less than 1% of described insect species and act as key ecosystem engineers through their effects on soil structuring, organic matter decomposition, nutrient cycling, and water regulation (Black and Okwakol 1997; Bignell and Eggleton 2000; Jouquet et al. 2011, 2016; Eggleton 2020; Rosenberg et al. 2023). At the same time, approximately 185 of the more than 3000 described species are recognized as pests and 28 as invasive, collectively generating billions of dollars in agricultural and structural damage annually worldwide (Su and Scheffrahn 2000; Evans 2011; Rust and Su 2012; Evans et al. 2013; Krishna et al. 2013; Govorushko 2019). Their ecological importance, combined with their frequent association with anthropogenic environments, makes termites especially pertinent model organisms for investigating the ecological and evolutionary consequences of urbanization (McGeogh 1998; McIntyre 2000; Chowdhury et al. 2023). Nevertheless, termite population genetics has rarely been examined within urban‐forest frameworks, and little is known about how urbanization affects termite connectivity, dispersal, and colony organization.

The subterranean termite *Heterotermes tenuis* (Hagen, 1858) is a widespread Neotropical species and an emerging invasive species throughout the Caribbean, including the Lesser Antilles, Trinidad and Tobago, and Puerto Rico (Szalanski et al. 2004; Evans et al. 2013; Duquesne and Fournier 2025). Native to the Neotropics, the species causes damage to wooden structures and a wide variety of crops such as sugarcane, rubber, eucalyptus, pine, and cotton (Su and Scheffrahn 2000; Constantino 2002b). Its xylophagous lifestyle and tendency to nest within its food resources may facilitate passive dispersal via the transport of infested wood and plant materials, contributing to its spread across anthropogenic landscapes (Donovan et al. 2001; Evans et al. 2013). However, despite its economic relevance and expanding range (Duquesne and Fournier 2025), little is known about how *H. tenuis* responds ecologically and genetically to urbanization.

Understanding these responses requires considering not only population genetic diversity and connectivity, but also colony breeding structure. Termite nests can be classified as simple families headed by a single monogamous reproductive pair, extended families containing multiple related neotenic reproductives, or mixed families founded or maintained by multiple reproductives from different lineages (Vargo and Husseneder 2011). These breeding systems strongly influence effective dispersal, inbreeding levels, and patterns of genetic differentiation among colonies (Vargo and Husseneder 2011). Consequently, colony organization can provide important insights into the demographic processes underlying population genetic structure. For example, weak genetic differentiation among distant colonies may reflect extensive dispersal or human‐mediated transport, whereas clusters of genetically related neighboring colonies may result from localized colony budding or secondary colony foundation involving neotenic reproductives (Husseneder et al. 2012; Zhang and Evans 2017; Chouvenc et al. 2025).

Environmental context is expected to shape both dispersal and colony establishment processes (Fahrig 2001; Perdereau et al. 2015). In forested habitats, dispersal through alate flights may be constrained by dense vegetation and other physical barriers, potentially promoting local recruitment and spatial genetic structure. Urbanization may alter these dynamics through contrasting effects on connectivity. On one hand, habitat fragmentation can reduce connectivity among populations (Frankham 1996; Fahrig 2003), promoting genetic drift and increasing genetic differentiation between habitat patches (Bickel et al. 2006; Louy et al. 2007). On the other hand, urban environments may function as bridgeheads that promote dispersal through the transport of infested materials and the establishment of human‐mediated dispersal pathways (Lombaert et al. 2010; Booth 2024; Duquesne and Fournier 2024). As a result, urban populations may exhibit reduced genetic diversity and increased isolation, or alternatively maintain high levels of diversity despite pronounced habitat‐associated genetic structure. Distinguishing between these scenarios is particularly relevant for invasive species, whose establishment and spread often depend on interactions between anthropogenic transport, local dispersal, and colony breeding systems (Dronnet et al. 2005; Aluko and Husseneder 2007; Fougeyrollas et al. 2018).

In this study, we examine how urbanization shapes the population genetics of the invasive termite *H. tenuis* in its introduced range in Guadeloupe. Using newly developed polymorphic microsatellite markers, we compare genetic diversity, population structure, isolation‐by‐distance patterns, and colony family structure between urban and forest habitats across three independent site pairs. Specifically, we test whether urbanization (i) alters levels of genetic diversity within colonies, (ii) modifies genetic connectivity and differentiation among colonies, (iii) disrupts spatial genetic structure, and (iv) influences colony breeding organization. By integrating landscape genetics and colony‐level analyses, this study provides insights into the mechanisms through which urbanization shapes the ecology and spread of an invasive termite in tropical island ecosystems.

## 2 Methods

Our study used a replicated landscape genetic design to evaluate how urbanization influences genetic diversity, population structure, dispersal processes, and colony organization in the invasive termite *H. tenuis* (Figure 1). Consistent with predictions that urban environments may reshape gene flow without necessarily reducing neutral genetic variation, we explicitly separated analyses of (i) genetic diversity, (ii) habitat‐associated genetic structure, (iii) isolation‐by‐distance, and (iv) colony breeding system.

**FIGURE 1:**
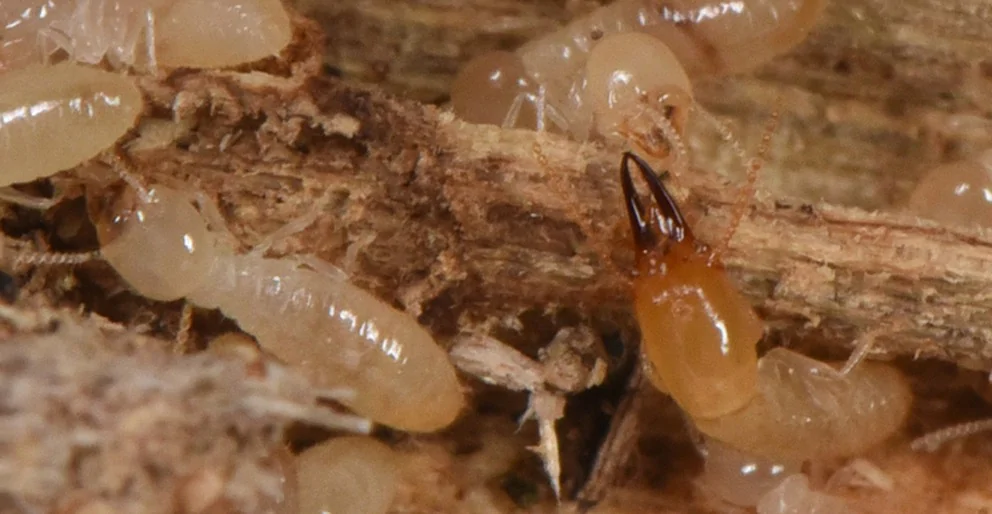
Soldier (right) and workers of *Heterotermes tenuis* . Photograph by Y. Roisin.

Sampling was conducted across three independent urban‐forest site pairs in Guadeloupe, enabling replication of habitat effects across spatially distinct landscapes and controlling for regional environmental heterogeneity.

### 2.1 Study Sites

Fieldwork was conducted in Guadeloupe (Lesser Antilles, Caribbean) in April–June 2023. *H. tenuis* is native to continental Central and South America and has been introduced to several Caribbean islands (Szalanski et al. 2004); all populations sampled here therefore belonged to the introduced range. Sampling followed a paired design comprising three urban‐forest pairs. Within each pair, an urban site was matched with the nearest suitable forest site to assess habitat‐associated differences while minimizing the influence of broad‐scale geographic variation. Forest sites were selected from the largest and best‐preserved fragment available in each landscape (> 1 km^2^), with sampling sites conducted at least 200 m from forest edges to avoid potential edge effects (Almeida et al. 2017). Urban habitats consisted of small city woodlots or wastelands embedded within urbanized matrices dominated by impervious surfaces. The paired urban and forest sites were separated by 4.7–11 km (Figure 2). Across the three pairs, urban sites had warmer soils (+3.2°C), higher night‐time light emission (+13.3 nW/cm^2^/sr), and higher human population density (+635 inhabitant/km^2^), whereas forest sites were located at higher elevation (+187 m) and had greater tree cover (+50%) (Table S1). A total of 50 *H. tenuis* nests were sampled across the three urban‐forest site pairs: Baie‐Mahault (5 forest and 5 urban nests), Petit‐Bourg (5 forest and 5 urban nests), and Sainte‐Rose (25 forest and 5 urban nests).

**FIGURE 2:**
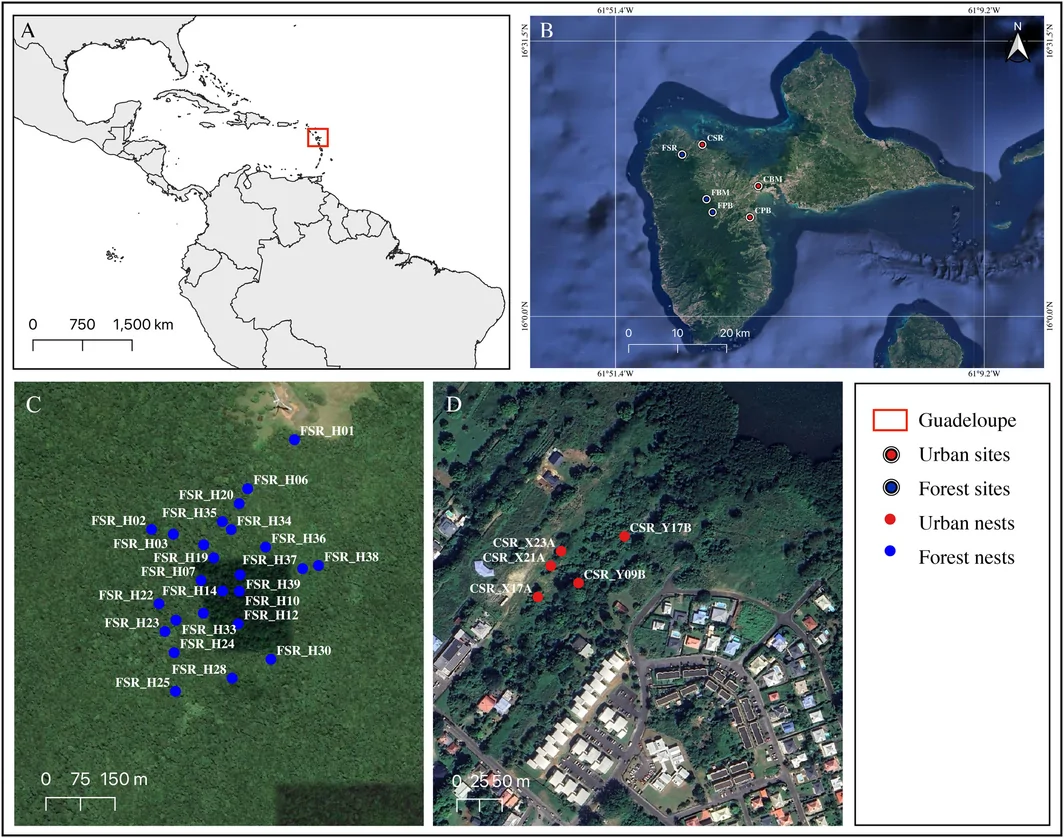
Location of sampling sites. (A) Caribbean region, (B) Guadeloupe (C = Urban, F = Forest), (C) Example of a forest site (FSR), (D) Example of an urban site (CSR). Maps were created using QGIS.

### 2.2 Nest Sampling and Biological Unit Definition

Sampling design differed slightly among sites because of habitat configuration. In the forest site of Sainte‐Rose (FSR), nests were searched for within a predefined sampling area using a random approach. In the other sites, nests were collected along transects at regular intervals. Nests were separated by at least 20 m, and median inter‐nest distances ranged from 53 to 200 m among sites. Because the foraging range of *H. tenuis* has not been quantified, spatial separation alone could not ensure that sampled nests represented independent colonies. Nest independence was therefore further evaluated using pairwise *F*_*CT*_ estimates and COLONY software 2.0.7.2 (Jones and Wang 2010). From each nest, 8–12 worker individuals were collected and preserved in 100% ethanol.

### 2.3 Species Identification and Molecular Confirmation

Specimens were first identified to genus using morphological keys (Constantino 2002a) and to species using region‐ and genus‐specific references (Constantino 2000). Species identity was further confirmed through DNA barcoding of the mitochondrial cytochrome oxidase II (COII) gene, a widely used marker for termite systematics (Ohkuma et al. 2004; Huang et al. 2011). DNA was extracted from worker head tissue using the NucleoSpin Tissue Kit (Macherey‐Nagel). A fragment of the COII gene (700–1000 bp) was amplified using the primers ModAtLeu (Miura et al. 2000) and B‐tLys (Simon et al. 1994). PCR products were purified and sequenced by capillary electrophoresis on an ABI 3730 DNA Analyzer (Applied Biosystems). Sequences were edited and aligned using CodonCode Aligner (CodonCode Corporation, www.codoncode.com) and compared against reference sequences available in GenBank using NCBI BLAST tool (NCBI 2023). All analyzed colonies were unambiguously assigned to *H. tenuis* .

### 2.4 Microsatellite Development

Species‐specific microsatellite loci were developed by Genoscreen (Lille, France). Total genomic DNA was extracted from 100 *H. tenuis* workers representative of the sampled populations using the NucleoSpin Tissue Kit (Macherey‐Nagel). An equimolar pool of genomic DNA (1 μg) was used to prepare a TruSeq Nano DNA library (Illumina), which was sequenced on an Illumina MiSeq platform using a Nano v2 reagent kit (2 × 250 bp paired‐end reads). Following demultiplexing and adapter trimming, paired‐end reads were merged using USEARCH (Edgar 2010). Because the number of merged sequences obtained after the initial processing was relatively low, an additional sequence recovery step was performed using PRINSEQ (Schmieder and Edwards 2011) with relaxed filtering parameters to maximize the number of informative reads available for marker discovery.

Microsatellite detection and primer design were conducted using QDD v3.1 (Meglécz et al. 2014). Sequencing generated 2,481,770 raw paired‐end reads (mean length = 310 bp), from which 45,542 merged sequences and 1,375,711 additional extended sequences were obtained after supplementary processing. QDD identified microsatellite‐containing sequences with di‐, tri‐, and tetranucleotide repeat motifs and designed corresponding primer pairs through its integrated Primer3 workflow (Rozen and Skaletsky 2000; Untergasser et al. 2012). After quality filtering, 5481 candidate primer pairs were retained for marker development.

From these candidates, 95 loci were selected for biological validation based on repeat motif characteristics, primer quality, and expected amplicon size. Amplification success was evaluated on six independent panels of eight workers representative of the studied populations. PCR products were screened using a QIAxcel Advanced System (Qiagen), and loci producing a specific amplicon of the expected size in at least five of the eight tested individuals were retained. This first screening step yielded 48 loci showing reliable amplification. The 48 retained loci were subsequently evaluated for polymorphism using an independent panel of 15 individuals. PCR products were analyzed on an ABI 3730XL DNA Analyzer (Applied Biosystems) using the GeneScan 500 LIZ size standard, and alleles were scored with GeneMapper v5.0 (Applied Biosystems). Based on amplification consistency, scoring reliability, and polymorphism, 20 loci were selected for multiplex development.

Multiplex combinations were initially designed using Multiplex Manager v1.2 (Holleley and Geerts 2009) and subsequently optimized empirically. Four multiplex PCR assays, each comprising five loci, were retained for population genetic analyses (Table S2). Primer concentrations were adjusted to balance amplification intensities while maintaining a common annealing temperature of 55°C.

### 2.5 Microsatellite Genotyping

A total of 414 workers from 50 nests of the three urban‐forest site pairs were genotyped at 20 microsatellite loci. Genomic DNA was extracted from individual worker heads using a modified CTAB protocol (Doyle and Doyle 1987). Microsatellite amplification was performed using four optimized multiplex PCR assays (Table S2). Forward primers were labeled with one of four fluorescent dyes (6‐FAM, VIC, NED, and PET) to allow simultaneous allele detection.

PCR amplifications were carried out in 20 μL reaction volumes containing approximately 5 ng of genomic DNA, 1× multiplex PCR master mix (2× Taq PCR Master Mix (Genesee Scientific)), 10 μM of each primer, and molecular‐grade water. Thermal cycling conditions consisted of an initial denaturation at 95°C for 3 min, followed by 35 cycles of denaturation at 95°C for 30 s, annealing at 55°C for 30 s, and extension at 72°C for 30 s, with a final extension step of 72°C for 5 min.

Amplified fragments were separated by capillary electrophoresis on an ABI 3730xl Genetic Analyzer (Applied Biosystems) using the GeneScan‐500LIZ standard (Applied Biosystems). Alleles were scored automatically and subsequently checked manually using STRyper version 1.3.1 (Peccoud 2025) to ensure consistency among electropherograms.

### 2.6 Genetic Analyses

All analyses were performed using R 4.1.2 (R Core Team 2021), and structured to explicitly test three non‐mutually exclusive processes identified in the study framework: (i) habitat effects on genetic diversity, (ii) habitat versus distance effects on genetic structure, and (iii) colony‐level breeding organization.

#### 2.6.1 Genotyping Quality Control and Marker Validation

Microsatellite data were screened for genotyping errors, including null alleles, stuttering and large‐allele dropout using MICRO‐CHECKER 2.2.3 (Van Oosterhout et al. 2004). Null allele frequencies were estimated per locus using the expectation–maximization algorithm implemented in FreeNA (Chapuis and Estoup 2007).

To ensure that population differentiation estimates were not biased by null alleles, global and locus‐specific *F*_*CT*_ (Thorne et al. 1999) values were compared with and without ENA correction. Consistency between corrected and uncorrected estimates supported retention of all loci for downstream inference.

#### 2.6.2 Genetic Diversity Within Colonies

Genetic diversity was quantified at the nest level using the 18 retained microsatellite loci. For each nest, allelic richness (*Ar*), observed heterozygosity (*Ho*), expected heterozygosity (*He*), and the inbreeding coefficient (*F*_*IC*_; Thorne et al. 1999) were calculated using the packages *adegenet* 2.1.10 (Jombart 2008) and *hierfstat* 0.5–11 (Goudet 2005). Differences in genetic diversity between urban and forest habitats were tested using linear mixed‐effects models (LMMs), fitted with habitat as a fixed effect. To account for the hierarchical structure of the sampling design, random intercepts were included for locus, nest, site, and site pair. Additional models were fitted separately for each urban‐forest site pair to test whether habitat effects were context‐dependent across landscapes. Likelihood‐ratio tests were used to assess the significance of fixed effects.

### 2.7 Population Genetic Structure and Habitat Differentiation

Population structure was assessed using Wright's *F*‐statistics (*F*_*IC*_, *F*_*CT*_, and *F*_*IT*_; Thorne et al. 1999), estimated with *hierfstat*. Confidence intervals (95%) were obtained by bootstrap resampling across loci. Genetic differentiation among nests was quantified using pairwise Weir and Cockerham's (1984) *F*_*CT*_ estimates. Pairwise *F*_*CT*_ matrices were visualized as heatmaps, and hierarchical clustering was used to identify patterns of genetic similarity among nests.

Multivariate patterns of genetic structure were explored by principal coordinates analyses (PCoA) based on pairwise genetic differentiation among nests. Habitat‐associated differences in genetic composition were tested separately within each urban‐forest pair using permutational multivariate analysis of variance (PERMANOVA; adonis2 function; 9999 permutations). Homogeneity of multivariate dispersions was evaluated with *betadisper* (*vegan* 2.6‐4 package) prior to interpreting PERMANOVA results.

A global PCoA and PERMANOVA were additionally performed using all six sampling sites. Site pair and habitat were included as explanatory factors, and their interaction was tested to determine whether the effect of habitat on genetic composition was consistent across the three landscapes. PERMANOVA terms were evaluated using both sequential and marginal tests. Homogeneity of multivariate dispersions was assessed for both site pair and habitat using *betadisper*.

#### 2.7.1 Isolation by Distance Versus Habitat Effects

Isolation by distance (IBD) was assessed using pairwise genetic and geographic distances among nests. Genetic distances were calculated as *F*_*CT*_/(1 − *F*_*CT*_) following Rousset (1997), and geographic distances were computed from nest coordinates using the *geosphere* 1.5–20 package (Hijmans 2024). Mantel tests (9999 permutations) were performed separately for each study site to test for correlations between geographic and genetic distances.

To account for the nonindependence of pairwise comparisons, Maximum Likelihood Population Effects (MLPE) models (Van Strien et al. 2012) were fitted using pairwise genetic differentiation as the response variable. Log‐transformed geographic distance and habitat comparison category (within urban, within forest, or between habitats) were included as fixed effects. Random intercepts were specified for site pair and for each nest involved in a comparison.

#### 2.7.2 Colony Breeding Structure

Family structure was inferred from worker microsatellite genotypes and classified into three categories following the criteria of Vargo et al. (2003). Simple families were headed by a single monogamous reproductive pair and exhibited no more than four alleles and four genotypes at any locus. Extended families were headed by multiple related neotenic reproductives produced within the nest and displayed no more than four alleles but more than four genotypes at one or more loci. Mixed families were founded or maintained by multiple unrelated reproductives and exhibited more than four alleles at one or more loci.

Colonies were classified as simple families, extended families, or mixed families based on the number of alleles and multilocus genotypes observed at each locus. Colonies exhibiting more than four alleles at one or more loci were classified as mixed families. For the remaining colonies, likelihood‐ratio *G*‐tests were used to compare observed genotype frequencies with Mendelian expectations under a simple‐family model headed by a single reproductive pair. The parental genotype combination minimizing the cumulative *G* statistic across loci was identified, and locus‐specific *G* statistics were combined into a global test for each colony. Family assignments were further evaluated using COLONY. Final classifications were established by combining evidence from allele counts, genotype diversity, *G*‐tests, and COLONY reconstructions.

## 3 Results

### 3.1 Genetic Diversity of *H. tenuis*

Of the 20 loci initially selected, two loci (*Hten‐09* and *Hten‐26*) were excluded from subsequent analyses because of poor amplification success and high levels of missing data (8.2% and 50% of genotypes obtained, respectively, compared to 100% for the 18 retained markers). Micro‐Checker and FreeNA detected no evidence of null alleles across loci. Estimated null allele frequencies were low overall (median *r̂* < 0.05), with a few loci showing moderate values in isolated populations (maximum *r̂* = 0.20); but no locus exhibited consistently elevated *r̂* across sites. Global estimates of genetic differentiation were nearly identical before and after ENA correction (*F*_*CT*_ = 0.144 vs. *F*_*CT*_‐ENA = 0.137), with per‐locus differences remaining small (∆*F*_*CT*_ < 0.02) and broadly overlapping 95% bootstrap confidence intervals. All 18 loci were therefore retained for subsequent analyses.

Pairwise *F*_*CT*_ values were similar for nests separated by < 100 m and more distant nests (median = 0.221 and 0.233, respectively), and 348 of 350 within‐site nest pairs had *F*_*CT*_ > 0.05. COLONY reconstructions assigned workers from the two pairs of nests with the smallest spatial separation to distinct full‐sib clusters, supporting their treatment as separate colonies. Each nest was therefore considered an independent colony unit.

A total of 414 workers from 50 nests were genotyped at 18 microsatellite loci, including 121 individuals from urban habitats and 293 from forest habitats. All loci were polymorphic, with 30 to 51 alleles per nest. Across loci, 65 alleles were shared between habitats, while 14 were found exclusively in urban habitats and 21 in forest habitats.

Genetic diversity was broadly similar between habitats (Figure 3; Table S3). Mean allelic richness ranged from 2.11 ± 0.26 in forest habitats to 2.35 ± 0.26 in urban habitats. Mean observed heterozygosity was higher than expected heterozygosity in both habitat types, averaging 0.47 ± 0.10 and 0.38 ± 0.07 in forest nests, and 0.56 ± 0.06 and 0.46 ± 0.05 in urban nests, respectively. Estimates of genetic differentiation among nests were similar between habitats, with mean *F*_*CT*_ values of 0.255 (95% CI: 0.224–0.292) in forest habitats and 0.243 (95% CI: 0.213–0.275) in urban habitats.

**FIGURE 3:**
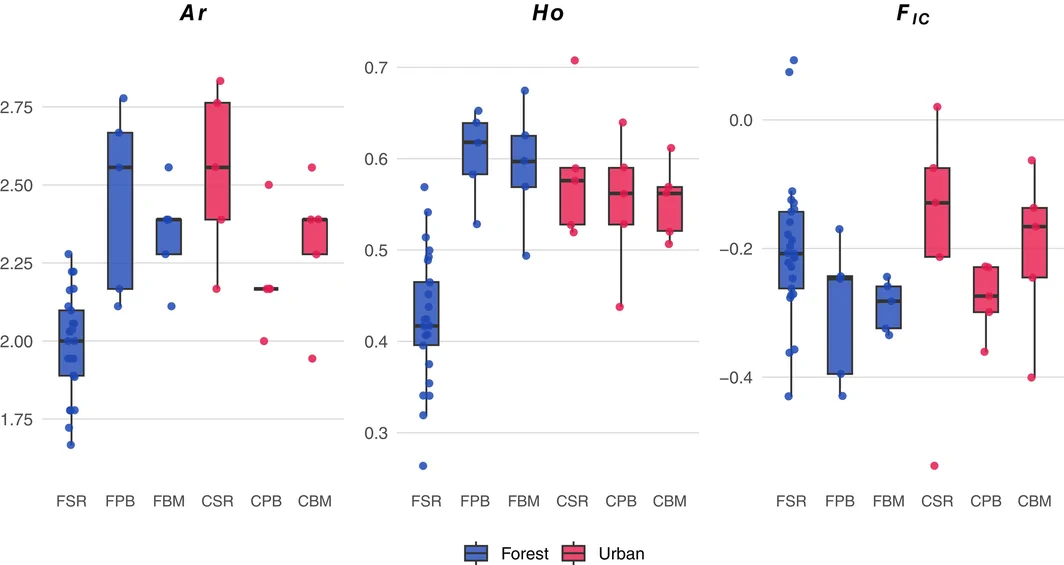
Distribution of allelic richness (*Ar*), observed heterozygosity (*Ho*), and inbreeding coefficient (*F*_*IC*_) among nests from forest and urban sites.

Linear mixed‐effects models revealed no overall effect of habitat on allelic richness (*Ar*), expected heterozygosity (*He*), observed heterozygosity (*Ho*), or the inbreeding coefficient (*F*_*IC*_) after accounting for the hierarchical structure of the sampling design (pair, site, nest, and locus). Habitat explained little variation in any genetic diversity indices, with nonsignificant effects for *Ar* (estimate = +0.098, *χ* ^*2*^ = 0.399, *p* = 0.528), *He* (estimate = +0.039, *χ* ^*2*^ = 0.995, *p* = 0.319), *Ho* (estimate = +0.055, *χ* ^*2*^ = 0.294, *p* = 0.588), and *F*_*IC*_ (estimate = +0.025, *χ* ^*2*^ = 0.679, *p* = 0.410) (Table S4).

In contrast, pair‐specific analyses revealed heterogeneous responses among site pairs. In the SR pair, urban nests exhibited significantly higher genetic diversity than forest nests, with increases of 0.552 alleles per locus for *Ar* (*p* < 0.001), 0.142 for *He* (*p* < 0.001), and 0.160 for *Ho* (*p* < 0.001). No habitat effect was detected for *F*_*IC*_. By contrast, none of the genetic diversity indices differed between habitats in either the PB and BM pairs (all *p* > 0.14).

### 3.2 Population Structure

Pairwise *F*_*CT*_ values among nests indicate substantial genetic differentiation among colonies (Figure 4). Hierarchical clustering of pairwise *F*_*CT*_ matrices revealed contrasting patterns among site pairs. In the SR pair, urban and forest nests formed distinct clusters. In the PB pair, nests from both habitats were intermixed, indicating no clear habitat‐associated structuring. The BM pair showed an intermediate pattern, with some clustering by habitat but without complete separation between urban and forest nests (Figure 3).

**FIGURE 4:**
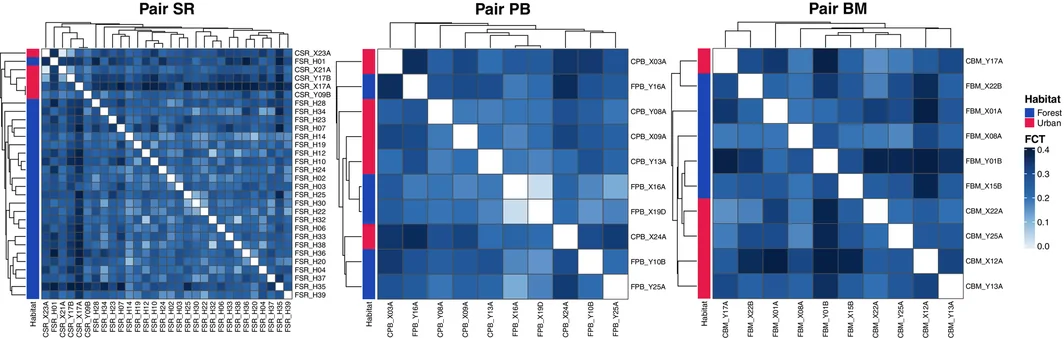
Heatmaps of pairwise *F*_*CT*_ values among nests in the three urban‐forest site pairs (SR, PB and BM). Nests are ordered according to hierarchical clustering based on pairwise *F*_*CT*_ values, with color intensity indicating the magnitude of differentiation. Row annotations indicate habitat (urban or forest).

Principal coordinate analysis (PCoA) further revealed habitat‐associated differentiation in all three site pairs (Figure 5). The first two axes explained 25.3%, 35.8%, and 45.8% of the total variation for the SR, PB, and BM pairs, respectively.

**FIGURE 5:**
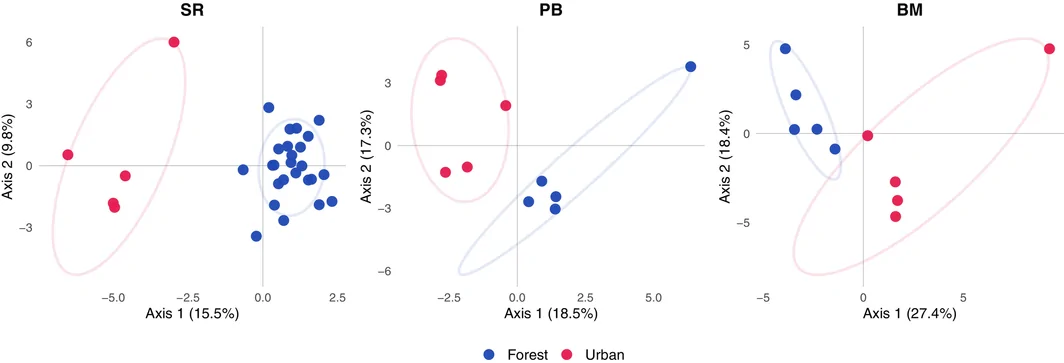
Principal coordinates analyses (PCoA) of nest‐level genetic differentiation performed separately for the three urban‐forest site pairs (SR, PB, and BM). Points represent individual nests and colors indicate habitat type. Ellipses correspond to 68% confidence intervals around habitat centroids. Percentages on the axes indicate the proportion of variation explained by each axis.

PERMANOVA analyses confirmed significant differences in genetic structure between urban and forest habitats within each site pair. Habitat explained 14.4%, 17.0%, and 21.5% of the total genetic variation in the SR, PB, and BM pairs, respectively (SR: *F* = 4.70, *R* ^*2*^ = 0.144, *p* < 0.001; PB: *F* = 1.64, *R* ^*2*^ = 0.170, *p* = 0.009; BM: *F* = 2.19, *R* ^*2*^ = 0.215, *p* = 0.007). Tests of multivariate homogeneity of dispersion were not significant for any pair (all *p* > 0.15), indicating that the observed differences reflected shifts in genetic structure rather than differences in within‐habitat variability.

In addition, a global PCoA including all 50 nests showed separation between forest and urban nests along the first axis, although the two habitat groups partially overlapped (Figure 6). Global PERMANOVA detected significant effects of both site pair (*F* = 10.08, *R* ^*2*^ = 0.263, *p* < 0.001) and habitat (*F* = 10.61, *R* ^*2*^ = 0.138, *p* < 0.001). Because habitat and site pair were spatially structured, both terms were evaluated using sequential and marginal tests. Habitat explained 13.8% of the total genetic variation in both analyses, indicating that the habitat effect was not accounted for by differences among site pairs. In contrast, the variation associated with site pair decreased from 26.3% in the sequential analysis to 15.9% in the marginal analysis, indicating that part of the variation associated with site pair was shared with habitat. The site pair × habitat interaction was not significant (*F* = 1.39, *R* ^*2*^ = 0.036, *p* = 0.155), indicating no detectable difference in the magnitude of habitat‐associated genetic differentiation among site pairs. Multivariate dispersion did not differ among habitats (*p* = 0.710) or site pairs (*p* = 0.373). To assess whether the unequal number of nests among sites, particularly the larger sample from the Sainte‐Rose forest site, influenced the result, PERMANOVA was repeated across 999 random subsamples of five nests per site. The habitat effect was significant in all 999 permutations (*p* < 0.05), with a median *R* ^*2*^ of 0.198 (2.5th–97.5th percentiles: 0.179–0.218).

**FIGURE 6:**
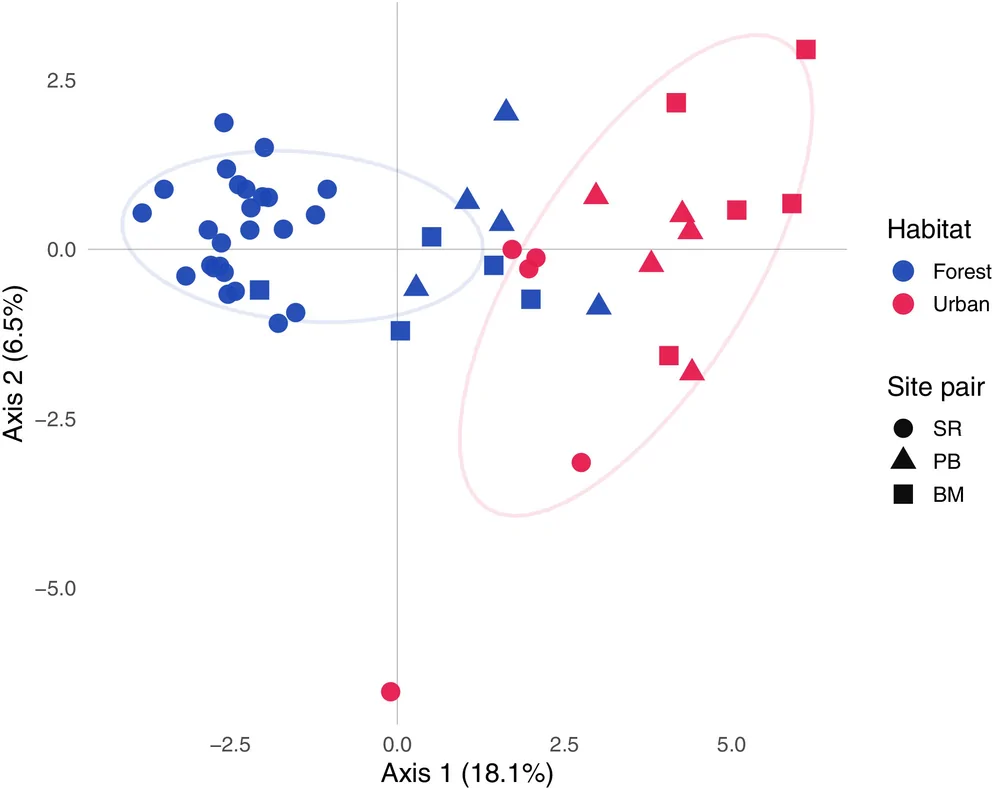
Principal coordinate analysis (PCoA) of genetic differentiation among the 50 *H. tenuis* nests sampled across the six study sites. Points represent individual nests and colors indicate habitat type. Ellipses correspond to 68% confidence intervals around habitat centroids. Percentages on the axes indicate the proportion of variation explained by each axis.

### 3.3 Isolation by Distance

Mantel tests detected a significant positive relationship between geographic and genetic distance only in the forest site FBM (Mantel *r* = 0.600, *p* = 0.033). No isolation‐by‐distance pattern was observed in any other forest or urban sites (all *p* > 0.156; Figure 7). These results indicate that geographic distance was not a consistent driver of genetic differentiation across the study area.

**FIGURE 7:**
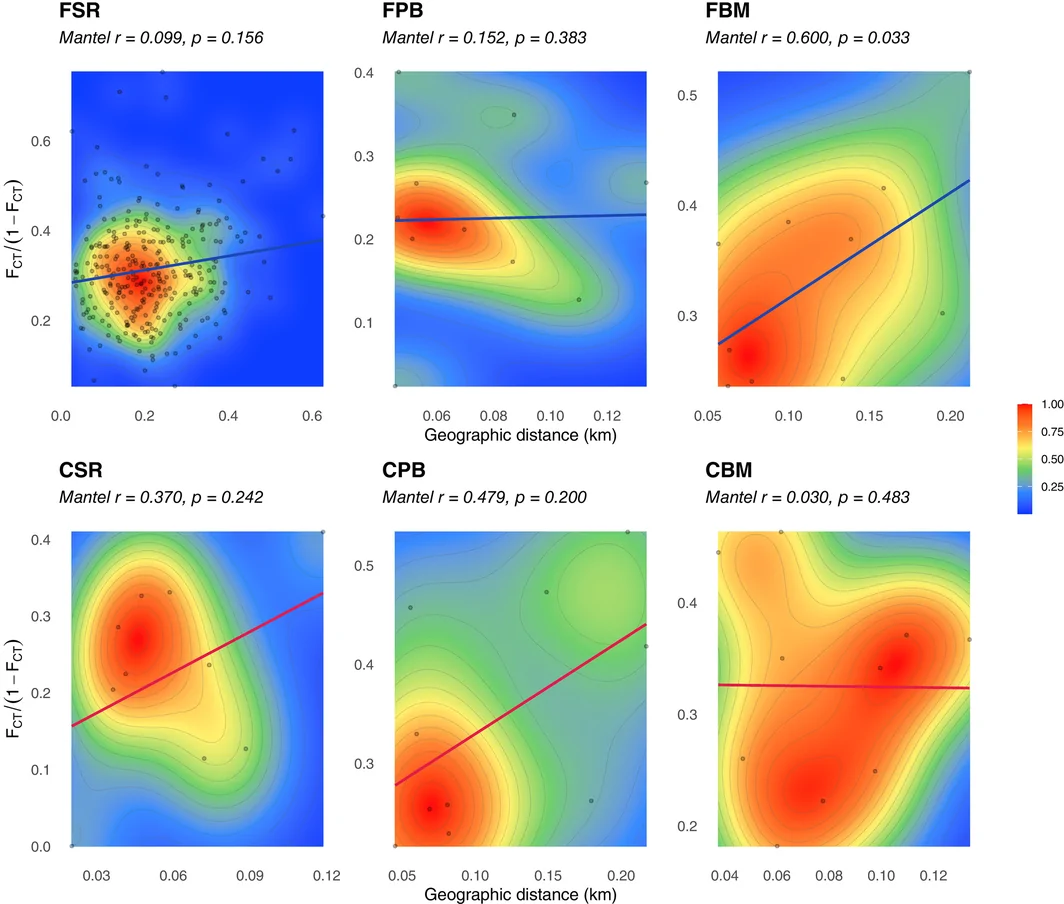
Relationships between geographic distance (log‐transformed km) and genetic differentiation (*F*_*CT*_/(1 − *F*_*CT*_)) among *H. tenuis* nests within each urban and forest site. Lines represent linear regressions and Mantel test statistics are indicated for each site. Color gradients represent point density.

Consistent with these results, the global MLPE model detected no effect of geographic distance on genetic differentiation (*ß* = 0.001 ± 0.009 SE, *p* = 0.878). In contrast, comparisons between urban and forest nests exhibited significantly greater genetic differentiation than comparisons within habitats (*ß* = 0.128 ± 0.042 SE, *p* = 0.002) (Table S5).

### 3.4 Family Structure

No mixed‐family colonies were detected among the 50 nests analyzed. Most nests were classified as extended families (37 nests, 74%), whereas only 13 nests (26%) corresponded to simple families. Extended‐family nests predominated in both forest (28 of 35 nests) and urban habitats (9 of 15 nests), and their frequency did not differ between habitats (Fisher's exact test, *p* = 0.170).

## 4 Discussion

Using newly developed microsatellite markers, we investigated how urbanization and associated habitat differences relate to genetic diversity, population structure, dispersal patterns, and colony organization in the invasive termite *H. tenuis* across paired urban and forest habitats in Guadeloupe. Contrary to the expectation that habitat fragmentation reduces genetic diversity through increased isolation and genetic drift, we found no evidence for a general reduction in allelic richness, heterozygosity, or inbreeding in urban populations. Nevertheless, urban and forest habitats consistently differed in genetic composition across the three site pairs, indicating that habitat context is associated with population genetic structure even when overall levels of genetic diversity are maintained. Together, these results suggest that urbanization is not necessarily associated with genetic erosion in this invasive termite, but rather with changes in the spatial organization of genetic variation.

### 4.1 Urban Population Maintain Genetic Diversity Despite Habitat Modification

A common prediction of urban landscape genetics is that habitat fragmentation reduces effective population size and restricts gene flow, ultimately leading to losses of genetic diversity through increased genetic drift (McKinney 2006, 2008; Elmqvist et al. 2013; Seto et al. 2013). Our results do not support this prediction. Allelic richness, observed heterozygosity, expected heterozygosity, and inbreeding coefficient were broadly similar between urban and forest habitats, and significant habitat effects were detected only in the Sainte‐Rose site pair, where urban nests showed higher allelic richness and observed heterozygosity than forest nests.

The absence of a consistent decline in genetic diversity suggests that urban populations of *H. tenuis* have not experienced the demographic bottlenecks often associated with habitat fragmentation. Several nonexclusive mechanisms may explain this pattern. First, urban populations may remain sufficiently connected to neighboring forest populations to maintain gene flow (Menke et al. 2010). Second, repeated colonization events, potentially facilitated by human activities, may continually introduce genetic variation into urban habitats (Estoup et al. 2016). Third, effective population sizes may remain large despite habitat modification because remnant habitat patches within the urban matrix can maintain sufficient local population sizes when some degree of connectivity persists (Nathan et al. 2017; de Sá Andrade et al. 2026; Sorrentino and Helms Cahan 2026).

Similar patterns have been reported in other social insects. Sorrentino and Helms Cahan (2026) found no evidence of reduced genetic diversity in urban ant populations relative to nearby forest populations, despite habitat fragmentation. Likewise, Khimoun et al. (2020) reported comparable levels of genetic diversity and weak genetic structuring between urban and forest populations of the acorn ant *Temnothorax nylanderi* (Foerster, 1850), while de Sá Andrade et al. (2026) observed that urban populations of the termite *Nasutitermes corniger* (Motschulsky, 1855) maintained levels of genetic diversity similar to those expected in less disturbed habitats. Together, these studies suggest that urbanization does not necessarily lead to neutral genetic impoverishment when populations remain demographically large or when some degree of connectivity persists between habitat patches (Nathan et al. 2017; Sorrentino and Helms Cahan 2026).

### 4.2 Habitat‐Associated Genetic Structure Emerges Despite Maintained Diversity

Although genetic diversity was largely unaffected by habitat type, analyses of population structure revealed a more complex picture. PERMANOVA and PCoA analyses consistently detected significant differences in genetic structure between urban and forest habitats within all three site pairs, while pairwise *F*_*CT*_ values indicated substantial differentiation among colonies. Habitat‐associated clustering was particularly pronounced in the SR pair, whereas PB and BM exhibited weaker but still detectable differentiation.

This combination of preserved genetic diversity and habitat‐associated structure suggests that differentiation can emerge without substantial genetic erosion. Genetic diversity and population structure reflect different demographic processes and can respond on different timescales: moderate restrictions to dispersal or differences in colony establishment and persistence may generate genetic differentiation among habitats before measurable losses of genetic diversity occur. However, the present results should not be interpreted as evidence that urbanization itself is the sole cause of this differentiation. Urban and forest sites differed in several environmental and landscape characteristics, including elevation, temperature, artificial light, human population density, and tree cover. These factors are intrinsically correlated with the urban‐forest contrast in our paired design and cannot be separated statistically using habitat as a binary predictor. In particular, environmental differences associated with elevation and temperature could potentially contribute to genetic differentiation through habitat‐dependent selection. Because the microsatellite markers used here are assumed to be selectively neutral, they primarily capture neutral genetic structure and cannot establish whether the observed differentiation has an adaptive component. Genome‐wide data combined with environmental association analyses and phenotypic measurements would be required to test this possibility.

Sampling effort was unbalanced, with a greater representation of forest nests from Sainte‐Rose. This reflected field constraints, as the number of nests available differed among sites despite comparable search effort. The unequal sampling effort did not appear to account for the observed habitat‐associated genetic structure, which remained consistent after controlling for sample size.

An alternative, non‐mutually exclusive explanation is that habitat filtering influences colony establishment success. In this scenario, dispersal may occur across habitat boundaries, but colonies founded in urban and forest environments experience different probabilities of survival and persistence (Grella et al. 2026). Consequently, observed genetic differentiation may reflect variation in establishment dynamics as much as variation in dispersal itself, although our data cannot distinguish between these processes.

Historical population structure provides another possible explanation. Genetically differentiated lineages may have been introduced independently into urban and forest environments and subsequently remained only partially mixed. Under such a scenario, the genetic differentiation detected between habitats would not necessarily result from contemporary urban landscape effects, but could instead reflect differences established before or during colonization of Guadeloupe (Facon et al. 2006). Because all populations sampled here originate from the introduced range and native‐range populations were not included, the present dataset cannot distinguish historical introduction structure from contemporary habitat‐associated processes.

These findings therefore challenge a simple view of cities as impermeable barriers to movement. Instead, urban landscapes may function as ecological stepping stones in which green spaces, remnant forest patches, gardens, and wooded corridors maintain sufficient connectivity to prevent genetic erosion while still promoting habitat‐associated structuring (Enedino et al. 2018; Chaves et al. 2026). Whether this structure primarily reflects landscape‐dependent dispersal, differential establishment, environmental selection, or historical population structure remains to be determined.

### 4.3 Habitat‐Associated Structure Is Stronger Than Geographic Distance

Further support for habitat‐associated processes comes from the isolation‐by‐distance analyses. Geographic distance explained little variation in genetic differentiation, with significant isolation by distance detected in only one of the six study sites. Consistent with this pattern, the MLPE model identified habitat context as a stronger predictor than geographic distance. Together, these results suggest that, at the spatial scale investigated, genetic connectivity in *H. tenuis* is shaped more strongly by environmental context than by simple spatial separation.

The weak and inconsistent isolation‐by‐distance patterns further suggest that processes other than dispersal through nuptial flights alone may contribute to connectivity. Connectivity may instead reflect a combination of natural dispersal and landscape‐dependent processes. One plausible mechanism is human‐mediated transport, which has been shown to play a major role in the spread of several invasive termites. In *Reticulitermes flavipes* (Kollar, 1837), the movement of infested wood, ornamental plants, and construction materials generates long‐distance jump dispersal events that weaken the relationship between genetic and geographic distance (Eyer et al. 2021). This process of stratified dispersal combines local biological dispersal through swarming or colony budding with stochastic long‐distance transport mediated by human activities (Shigesada et al. 1995; Eyer et al. 2021).

A similar mechanism could contribute to the patterns observed in *H. tenuis* . Because colonies nest within their food resources, infested wood and plant materials can be transported inadvertently over distances far exceeding those achievable through natural dispersal alone. Human‐mediated transport could therefore help maintain connectivity among urban populations while weakening isolation‐by‐distance patterns. This interpretation is consistent with the maintenance of genetic diversity despite significant habitat‐associated genetic structuring, suggesting that urban landscapes may simultaneously facilitate long‐distance dispersal and promote local genetic differentiation. However, this scenario remains hypothetical and was not tested directly. Distinguishing human‐mediated transport from natural dispersal would require sampling along potential transport corridors and explicit modeling of invasion pathways.

These conclusions apply primarily to the spatial scale sampled here, spanning within‐site distances and paired urban‐forest sites. Whether the same pattern extends across Guadeloupe and to the larger spatial scales relevant to human‐mediated transport remains unknown. Although the confidence interval of the distance coefficient excludes a strong effect of geographic distance, it remains compatible with a moderate effect. Thus, our results suggest that habitat‐associated differentiation was stronger than the effect of geographic distance at the scale investigated, rather than that geographic distance is irrelevant. Moreover, because urban and forest sites differed in several correlated environmental and landscape characteristics, the contribution of specific components of the urban environment cannot be identified from the present binary habitat classification. Quantifying these characteristics along a continuous urbanization gradient and at the nest level would help identify which aspects of the urban environment contribute most to the observed genetic structuring.

### 4.4 Colony Breeding Structure Appears Robust to Urbanization

Colony breeding structure appeared relatively stable across urban and forest habitats. Extended families predominated in both environments, and no mixed‐family colonies were detected. To our knowledge, colony breeding structure has not previously been characterized in *Heterotermes*. Across the Rhinotermitidae, the prevalence of extended families varies among species and populations. In native *R. flavipes* , extended families account for 15%–74% of colonies depending on the population, with similarly broad variation reported in *Reticulitermes grassei* (Clément, 1977) (Vargo and Husseneder 2011). In introduced *Coptotermes formosanus* (Shiraki, 1909), simple families generally predominate (Vargo et al. 2003, 2006; Eyer and Vargo 2021), whereas introduced *R. flavipes* and *Reticulitermes urbis* (Bagnères, Uva and Clément, 2003) are composed almost exclusively of extended and mixed families (Dronnet et al. 2005; Leniaud et al. 2010). The 74% prevalence of extended families observed here therefore falls at the upper end of the range documented for native populations, without reaching the near‐exclusive dominance of extended families reported in some introduced populations. Whether this pattern is associated with the introduced status of *H. tenuis* in Guadeloupe cannot be assessed because all populations sampled here belong to the introduced range.

Our results suggest that urban populations of *H. tenuis* do not rely on fundamentally different reproductive strategies to persist in anthropogenic environments. Instead, urban colonies appear to retain breeding systems already present in non‐urban populations. Similar patterns have been reported for other social insects, where colony structure often reflects intrinsic species biology rather than direct response to urbanization. Menke et al. (2010) showed that colony structure in *Tapinoma sessile* (Say, 1836) was not strictly associated with urban habitats, with both monogynous and polygynous colonies occurring across habitat types, and concluded that reproductive strategy reflects a plastic trait rather than a habitat‐specific adaptation. Likewise, de Sá Andrade et al. (2026) highlighted that high genetic connectivity does not necessarily preclude inbreeding, as colony breeding systems may strongly influence genetic structure independently of landscape connectivity.

Because breeding structure strongly influences effective dispersal, relatedness patterns, and within‐population genetic variance (Ross 2001; Vargo 2019; Eyer and Vargo 2021), the predominance of extended families may also contribute to the substantial differentiation observed among neighboring colonies. Consequently, colony‐level processes likely interact with landscape‐level processes and should be considered jointly when interpreting patterns of genetic structure in termites.

### 4.5 Implications for Invasion Dynamics

A limitation of the present study is that all sampled populations originated from the introduced range of *H. tenuis* in Guadeloupe and that only three independent urban‐forest site pairs were investigated, limiting the generality of our conclusions across tropical urban ecosystems. Moreover, because no native‐range populations were sampled, the genetic diversity observed in Guadeloupe cannot be evaluated relative to that of potential source populations. The diversity maintained in the introduced range could reflect relatively limited genetic erosion following introduction, but could also result from multiple introductions from genetically divergent source populations, followed by admixture among introduced lineages (Kolbe et al. 2004; Frankham 2005; Daly et al. 2023). Similarly, historical differentiation between source lineages could contribute to the habitat‐associated structure observed if different lineages were introduced into urban and forest environments and subsequently remained only partially admixed. Thus, the present data cannot distinguish between alternative invasion histories and contemporary landscape processes that may generate similar patterns of neutral genetic diversity and structure within the introduced range.

In some invasive termites, such as *C. formosanus* , introduced populations exhibit reduced genetic diversity despite evidence of multiple introductions (Husseneder et al. 2012). In contrast, in *R. flavipes* , repeated introductions and admixture among divergent source populations can maintain or even increase genetic diversity in invaded ranges (Eyer et al. 2021). Similar processes could potentially contribute to the genetic diversity observed in *H. tenuis* in Guadeloupe, but distinguishing among these scenarios requires comparisons with populations from the native range and explicit inference of source populations and introduction pathways.

More broadly, our study contributes to a growing body of work in urban landscape genetics of invasive species (Fusco et al. 2021) by showing that anthropogenic landscapes can be associated with genetic structure without detectable losses of neutral genetic diversity. The observed habitat‐associated differentiation may arise from contemporary differences in dispersal or colony establishment, environmental differences associated with urbanization, historical introduction structure, or interactions among these processes. Disentangling their relative contributions will require integration of native‐range sampling to reconstruct invasion history, genome‐wide data to resolve population structure and test for adaptive differentiation, and explicit landscape‐genetic modeling to quantify the effects of environmental heterogeneity, urban configuration, and potential human‐mediated transport pathways.

In conclusion, urbanization was not associated with detectable losses of neutral genetic diversity in *H. tenuis* , but urban and forest habitats consistently differed in genetic structure. Habitat‐associated differentiation was stronger than the effect of geographic distance at the spatial scale investigated, while colony breeding organization remained largely unchanged across habitats. These findings suggest that habitat context can be associated with population genetic structure without substantial genetic erosion, potentially reflecting the combined influence of landscape‐dependent dispersal, habitat‐specific colony establishment, environmental heterogeneity, and historical introduction processes. This complexity highlights the importance of considering both landscape configuration and colony biology when assessing dispersal dynamics and monitoring the spread of invasive social insects in rapidly urbanizing tropical regions.

## Author Contributions

**Emilie Gheyle:** data curation (equal), formal analysis (equal), investigation (equal), software (equal), validation (equal), visualization (equal), writing – original draft (equal), writing – review and editing (equal). **Edouard Duquesne:** conceptualization (equal), data curation (equal), investigation (equal), methodology (equal), project administration (equal), writing – review and editing (equal). **Yves Roisin:** investigation (equal), methodology (equal), writing – review and editing (equal). **Denis Fournier:** conceptualization (equal), funding acquisition (equal), investigation (equal), methodology (equal), supervision (equal), writing – original draft (equal), writing – review and editing (equal).

## Funding

This work was supported by Fonds De La Recherche Scientifique ‐ FNRS, J.0063.21, J0193‐25, FRIA (E.D.), 2.5020.11; Université libre de Vruxelles, Fonds Defay (D.F.)

## Conflicts of Interest

The authors declare no conflicts of interest.

## Supporting information


**Table S1:** Characteristics of the three urban‐forest site pairs. Coordinates correspond to the geographic centre of each site, used as the reference point for distance calculations and for the extraction of environmental variables. Variables were extracted from the following sources: elevation, Amatulli et al. (2018); soil annual mean temperature, Lembrechts et al. (2022); fractional tree cover, Liu and Liu (2024); night‐time light emission, Elvidge et al. (2021); human population density, Wang et al. (2022).
**Table S2:** Microsatellite loci tested for *Heterotermes tenuis* , including primer sequences, repeat motifs, allele size ranges, and number of alleles per locus. The two loci with low success rates were excluded.
**Table S3:** Genetic diversity statistics estimated from 18 microsatellite loci for each *Heterotermes tenuis* nest, with site and habitat summaries. *n* = number of nests; *N* = number of genotyped workers. No. Alleles = total number of alleles observed across all loci within the nest. *Ar* = allelic richness (mean ± SD); *He* = expected heterozygosity (mean ± SD); *Ho* = observed heterozygosity (mean ± SD); *F*_*IC*_ = intra‐nest inbreeding coefficient; *F*_*IC*_ = overall inbreeding coefficient; *F*_*CT*_ = genetic differentiation of the nest relative to all other nests. *F*‐statistics reported as mean estimates with 95% bootstrap confidence intervals in parentheses. Site and habitat summary rows (bottom) report means ± SD across nests, with *F*‐statistics averaged across nests with 95% bootstrap confidence intervals. C = urban site; F = forest site.
**Table S4:** Results of linear mixed‐effects models (LMM) testing the effect of habitat type (urban vs. forest) on genetic diversity indices. Results are shown for the global model and for each pair separately. *Ar* = allelic richness; *He* = expected heterozygosity; *Ho* = observed heterozygosity; *F*_*IC*_ = intra‐nest inbreeding coefficient. Global model: estimate and SE of the habitat fixed effect; *χ* ^*2*^ and *p* from likelihood ratio tests (LRT, df = 1). Pair‐specific model: estimates and SE from emmeans contrasts (df = 44 for all comparisons); *p* (Bonferroni) = *p*‐values corrected across simultaneous comparisons. Bold values indicate *p* < 0.05.
**Table S5:** Results of the global MLPE model testing the effects of geographic distance and habitat comparison on pairwise genetic differentiation (*F*_*CT*_).

## Data Availability

The data that support the findings of this study are available in the Supporting Information.
