# Supplementary material for: Urban Habitat Mosaics Structure Gene Flow but Preserve Genetic Diversity in an Invasive Termite
Source: Ecol Evol. 2026 Oct 19;16(10):e74439. doi: 10.1002/ece3.74439 (PMC13680549; doi:10.1002/ece3.74439)
Supplement: Supplementary file 1 [file ECE3-16-e74439-s001.docx]

**Table S1.** Characteristics of the three urban-forest site pairs. Coordinates correspond to the geographic centre of each site, used as the reference point for distance calculations and for the extraction of environmental variables. Variables were extracted from the following sources: elevation, Amatulli *et al*. (2018); soil annual mean temperature, Lembrechts *et al*. (2022); fractional tree cover, Liu & Liu (2024); night-time light emission, Elvidge *et al*. (2021); human population density, Wang *et al*. (2022).

| **Paire** | **Site** | **Habitat** | **Lat** | **Long** | **Distance between urban and forest sites (km)** | **Altitude (m)** | **Soil annual mean temperature (°C)** | **Nightlight (nW/cm^2^/sr)** | **Fractional tree cover (%)** | **Human population** |
| --- | --- | --- | --- | --- | --- | --- | --- | --- | --- | --- |
| ***Sainte-Rose*** | CSR | Urban | 16.328 | -61.691 | 4.72 | 4.00 | 26.65 | 13.77 | 17 | 571.97 |
|  | FSR | Forest | 16.308 | -61.730 |  | 296.50 | 22.62 | 0.00 | 100 | 35.18 |
| ***Baie-Mahault*** | CBM | Urban | 16.248 | -61.584 | 11.00 | 27.50 | 26.76 | 19.59 | 34 | 858.46 |
|  | FBM | Forest | 16.224 | -61.684 |  | 171.00 | 23.84 | 0.00 | 96 | 26.14 |
| **Petit-Bourg** | CPB | Urban | 16.189 | -61.600 | 7.66 | 28.00 | 26.92 | 6.53 | 91 | 578.00 |
|  | FPB | Forest | 16.198 | -61.671 |  | 152.50 | 24.28 | 0.00 | 96 | 41.21 |

**Table S2.** Microsatellite loci tested for *Heterotermes tenuis*, including primer sequences, repeat motifs, allele size ranges, and number of alleles per locus. The two loci with low success rates were excluded.

| **Mix** | **Locus** | **5’-3’ sequence** | **Motif** | **Allele length (bp)** | **No. alleles** |
| --- | --- | --- | --- | --- | --- |
| **M1** | Hten-26 | F - ACGTTTCGATCCTCAACGCA | (AT)15 | 182, 184 | 2 |
|  |  | R - TCGAATACGAGCTTTGCACA |  |  |  |
|  | **Hten-28** | F - CTCGTCCCAGCTGCTGATAT | (AC)14 | 280, 282, 296, 298, 300, 308 | 6 |
|  |  | R - TGTCTCGCTTACATGCCAGT |  |  |  |
|  | **Hten-64** | F - AATTCGTTCACTGTACGCCA | (AT)12 | 178, 190, 192, 194, 196, 198, 206 | 7 |
|  |  | R - AGACCAACAATCAGGCACGA |  |  |  |
|  | **Hten-70** | F - ACTTACAAACCTTTCCAATCGATT | (AG)12 | 100, 102, 106, 118 | 4 |
|  |  | R - CCACGTCATCACAAACCACTG |  |  |  |
|  | **Hten-93** | F - GCCAGGAGGATGAAAGACGT | (AG)10 | 285, 289, 291, 293, 297 | 5 |
|  |  | R - TTTGCCACTCATCCCTGTCG |  |  |  |
| **M2** | **Hten-08** | F - CACAATAACGTGTCGTCGTGT | (ACT)18 | 139, 218, 236, 239, 242, 248 | 6 |
|  |  | R - TTCTGTGTTCCACTTGCATAGT |  |  |  |
|  | **Hten-24** | F - CCGCTGGACCTACTGCTATC | (AAT)15 | 175, 178, 184, 187, 214 | 5 |
|  |  | R - CCGATTTCGTCACAGACATGA |  |  |  |
|  | **Hten-42** | F - GCTTTGTGCAGTGTTAGAAATATTTG | (AT)14 | 151, 153, 155, 157, 159, 161 | 6 |
|  |  | R - CCGATGATGGCACGATCAGA |  |  |  |
|  | **Hten-81** | F - CACCCTATTGCCTGTCAGGT | (AT)11 | 214, 216, 218 | 3 |
|  |  | R - ATACCACCACGTAGTTGCGT |  |  |  |
|  | **Hten-90** | F - ACTATGCACATCAAGTTGCCC | (AC)11 | 122, 126, 128, 130, 134, 136, 140, 146 | 8 |
|  |  | R - TGTTCACCATACACAGGCCC |  |  |  |
| **M3** | Hten-09 | F - ACCTTTATGTCTACATATGTGAACGA | (AAT)18 | 206, 215, 227 | 3 |
|  |  | R - GTCTCGTTGTGAACAAATTGCT |  |  |  |
|  | **Hten-10** | F - AAATTCACCTCCTGTCCACCA | (AC)17 | 290, 298, 300, 302 | 4 |
|  |  | R - CCCGCAGCAAACTGGAATAC |  |  |  |
|  | **Hten-45** | F - GGCAATGAATGTCGATCGCT | (ACT)13 | 281, 284, 287, 293 | 4 |
|  |  | R - GCCCTTCGCATTACGTCCAT |  |  |  |
|  | **Hten-69** | F - AAGTGTACGTTGCAGCTGGT | (AT)12 | 118, 124, 126, 128, 134, 138, 140, 146, 150 | 9 |
|  |  | R - CGAGTACCTTACTGCGTGCA |  |  |  |
|  | **Hten-87** | F - GTAAGACTACTGCCACCGGG | (AAT)11 | 136, 139, 157, 159, 160, 163 | 6 |
|  |  | R - GCTATCGCCCTGCCATCTAT |  |  |  |
| **M4** | **Hten-02** | F - TGGTTAGGGTTAGGTTCACAGT | (AT)24 | 122, 124, 126, 128, 130, 132, 134, 140 | 8 |
|  |  | R - AGGAAGGCTTTATAACATTCAGTTCA |  |  |  |
|  | **Hten-35** | F - ACTTCCCATTTCCGAGGTCT | (AC)14 | 197, 201, 203, 207, 209 | 5 |
|  |  | R - AAGTTGGACTGGAGAAGGCT |  |  |  |
|  | **Hten-46** | F - TAGTGAATACAAGAATGCAGGTTGT | (AC)13 | 189, 197, 203, 285, 287, 289, 298 | 7 |
|  |  | R - AAGAAATTTGCACACCCGGC |  |  |  |
|  | **Hten-49** | F - ATTTGCTCACAACCCTGAGGA | (AAC)13 | 230, 236, 239, 287 | 4 |
|  |  | R - AAATTGTGCATTTGGTTTGTCTTAAA |  |  |  |
|  | **Hten-71** | F - GGGCAGGGTAGAAAGAGAAACT | (ATC)11 | 290, 296, 299 | 3 |
|  |  | R - CGTTTCCGTGGGCTATTTGG |  |  |  |

**Table S3.** Genetic diversity statistics estimated from 18 microsatellite loci for each *Heterotermes tenuis* nest, with site and habitat summaries. *n* = number of nests; *N* = number of genotyped workers. No. Alleles = total number of alleles observed across all loci within the nest. *Ar* = allelic richness (mean ± SD); *He* = expected heterozygosity (mean ± SD); *Ho* = observed heterozygosity (mean ± SD); *F_IC_* = intra-nest inbreeding coefficient; *F_IC_* = overall inbreeding coefficient; *F_CT_* = genetic differentiation of the nest relative to all other nests. *F*-statistics reported as mean estimates with 95% bootstrap confidence intervals in parentheses. Site and habitat summary rows (bottom) report means ± SD across nests, with *F*-statistics averaged across nests with 95% bootstrap confidence intervals. C = urban site; F = forest site.

| **Site** | **Nest** | **Lat** | **Long** | **No. Alleles** | ***Ar*** | ***He*** | ***Ho*** | ***F_IC_*** | ***F_IT_*** | ***F_CT_*** | **Family Type** |  |
| --- | --- | --- | --- | --- | --- | --- | --- | --- | --- | --- | --- | --- |
| **Sainte-Rose  Urban Site  (CSR)** | CSR_X17A | 16.3285 | -61.6906 | 39 | 2.167 ± 0.514 | 0.453 ± 0.145 | 0.708 ± 0.288 | -0.538  (-0.678 – -0.407) | -0.323  (-0.707 – -0.014) |  | Simple |  |
|  | CSR_X21A | 16.3288 | -61.6905 | 50 | 2.763 ± 0.717 | 0.538 ± 0.122 | 0.519 ± 0.202 | 0.02  (-0. 127 – 0.179) | 0.013  (-0.262 – 0.245) |  | Extended |  |
|  | CSR_X23A | 16.3290 | -61.6904 | 46 | 2.556 ± 0.784 | 0.490 ± 0.195 | 0.528 ± 0.305 | -0.075  (-0.264 – 0.110) | 0.069  (-0.181 – 0.313) |  | Extended |  |
|  | CSR_Y09B | 16.3286 | -61.6902 | 51 | 2.833 ± 0.857 | 0.513 ± 0.238 | 0.590 ± 0.342 | -0.129  (-0.290 – 0.042) | 0.006  (-0.220 – 0.265) |  | Simple |  |
|  | CSR_Y17B | 16.3291 | -61.6897 | 43 | 2.389 ± 0.698 | 0.474 ± 0.177 | 0.576 ± 0.278 | -0.213  (-0.356 – -0.051) | -0.012  (-0.186 – 0.172) |  | Extended |  |
| **Sainte-Rose  Forest Site  (FSR)** | FSR_H01 | 16.3103 | -61.7285 | 37 | 2.031 ± 0.702 | 0.331 ± 0.218 | 0.407 ± 0.277 | -0.222  (-0.339 – -0.091) | 0.319  (0.138 – 0.526) |  | Extended |  |
|  | FSR_H02 | 16.3085 | -61.73144 | 41 | 2.278 ± 1.018 | 0.416 ± 0.252 | 0.465 ± 0.303 | -0.129  (-0.249 – 0.018) | 0.149  (-0.124 – 0.430) |  | Simple |  |
|  | FSR_H03 | 16.3084 | -61.7310 | 38 | 2.098 ± 0.564 | 0.405 ± 0.172 | 0.489 ± 0.237 | -0.212  (-0.323 – -0.084) | 0.098  (-0.173 – 0.334) |  | Simple |  |
|  | FSR_H04 | 16.3082 | -61.7303 | 40 | 2.222 ± 0.878 | 0.360 ± 0.235 | 0.424 ± 0.324 | -0.159  (-0.331 – 0.040) | 0.265  (0.026 – 0.508) |  | Extended |  |
|  | FSR_H06 | 16.3093 | -61.7294 | 40 | 2.163 ± 0.840 | 0.359 ± 0.264 | 0.417 ± 0.338 | -0.139 (-0.277 – 0.001) | 0.272  (-0.024 – 0.553) |  | Extended |  |
|  | FSR_H07 | 16.3075 | -61.7304 | 32 | 1.778 ± 0.548 | 0.305 ± 0.225 | 0.396 ± 0.338 | -0.273  (-0.469 – -0.077) | 0.286  (-0.021 – 0.568) |  | Extended |  |
|  | FSR_H10 | 16.3073 | -61.7296 | 34 | 1.885 ± 0.673 | 0.333 ± 0.232 | 0.417 ± 0.307 | -0.262  (-0.419 – -0.103) | 0.299  (0.060 – 0.523) |  | Extended |  |
|  | FSR_H12 | 16.3066 | -61.7297 | 34 | 1.889 ± 0.676 | 0.324 ± 0.223 | 0.417 ± 0.321 | -0.271  (-0.422 – -0.109) | 0.302  (0.056 – 0.539) |  | Simple |  |
|  | FSR_H14 | 16.3073 | -61.7300 | 40 | 2.222 ± 0.428 | 0.464 ± 0.132 | 0.514 ± 0.287 | -0.111  (-0.325 – 0.101) | 0.035  (-0.202 – 0.271) |  | Extended |  |
|  | FSR_H19 | 16.3079 | -61.7301 | 36 | 2.000 ± 0.686 | 0.395 ± 0.203 | 0.569 ± 0.313 | -0.430  (-0.568 – -0.312) | 0.033  (-0.211 – 0.293) |  | Extended |  |
|  | FSR_H20 | 16.3090 | -61.7296 | 37 | 2.056 ± 0.639 | 0.370 ± 0.219 | 0.417 ± 0.306 | -0.124  (-0.297 – 0.077) | 0.285  (0.066 – 0.516) |  | Extended |  |
|  | FSR_H22 | 16.3070 | -61.7312 | 35 | 1.944 ± 0.539 | 0.383 ± 0.206 | 0.451 ± 0.324 | -0.187  (-0.413 – 0.055) | 0.233  (-0.021 – 0.477) |  | Extended |  |
|  | FSR_H23 | 16.3064 | -61.7311 | 39 | 2.167 ± 0.857 | 0.390 ± 0.233 | 0.542 ± 0.371 | -0.362  (-0.535 – -0.194) | 0.111  (-0.148 – 0.377) |  | Extended |  |
|  | FSR_H24 | 16.3060 | -61.7309 | 37 | 2.056 ± 0.873 | 0.361 ± 0.268 | 0.438 ± 0.327 | -0.215  (-0.306 – -0.128) | 0.308  (0.078 – 0.540) |  | Simple |  |
|  | FSR_H25 | 16.3052 | -61.7309 | 30 | 1.667 ± 0.686 | 0.274 ± 0.256 | 0.340 ± 0.342 | -0.246  (-0.469 – -0.043) | 0.450  (0.193 – 0.688) |  | Simple |  |
|  | FSR_H28 | 16.3055 | -61.7298 | 38 | 2.111 ± 0.963 | 0.346 ± 0.270 | 0.417 ± 0.371 | -0.208  (-0.412 – 0.026) | 0.238  (-0.123 – 0.553) |  | Extended |  |
|  | FSR_H30 | 16.3059 | -61.7290 | 37 | 2.036 ± 0.249 | 0.408 ± 0.138 | 0.500 ± 0.248 | -0.196  (-0.346 – -0.045) | 0.059  (-0.159 – 0.275) |  | Extended |  |
|  | FSR_H32 | 16.3068 | -61.7303 | 35 | 1.944 ± 0.539 | 0.342 ± 0.206 | 0.319 ± 0.254 | 0.074  (-0.128 – 0.292) | 0.470  (0.289 – 0.649) |  | Extended |  |
|  | FSR_H33 | 16.3067 | -61.7309 | 37 | 2.030 ± 0.788 | 0.319 ± 0.265 | 0.407 ± 0.373 | -0.228  (-0.420 – -0.047) | 0.312  (0.030 – 0.595) |  | Extended |  |
|  | FSR_H34 | 16.3085 | -61.7298 | 35 | 1.944 ± 0.802 | 0.301 ± 0.251 | 0.354 ± 0.324 | -0.178  (-0.347 – 0.020) | 0.435  (0.189 – 0.655) |  | Extended |  |
|  | FSR_H35 | 16.3087 | -61.7300 | 31 | 1.722 ± 0.752 | 0.278 ± 0.246 | 0.34 ± 0.348 | -0.207  (-0.443 – 0.017) | 0.447  (0.192 – 0.688) |  | Extended |  |
|  | FSR_H36 | 16.3082 | -61.7291 | 32 | 1.778 ± 0.647 | 0.306 ± 0.245 | 0.424 ± 0.379 | -0.357  (-0.557 – -0.166) | 0.296  (-0.031 – 0.583) |  | Extended |  |
|  | FSR_H37 | 16.3077 | -61.7283 | 36 | 2.000 ± 0.97 | 0.333 ± 0.270 | 0.375 ± 0.300 | -0.143  (-0.241 – -0.055) | 0.383  (0.171 – 0.593) |  | Extended |  |
|  | FSR_H38 | 16.3078 | -61.7280 | 32 | 1.778 ± 0.647 | 0.303 ± 0.250 | 0.264 ± 0.277 | 0.093  (-0.194 – 0.400) | 0.581  (0.389 – 0.780) |  | Extended |  |
|  | FSR_H39 | 16.3076 | -61.7296 | 35 | 1.944 ± 0.539 | 0.379 ± 0.206 | 0.493 ± 0.344 | -0.277  (-0.498 – -0.059) | 0.182  (-0.073 – 0.442) |  | Extended |  |
| **Petit-Bourg  Urban Site  (CPB)** | CPB_X03A | 16.1888 | -61.6009 | 39 | 2.167 ± 0.618 | 0.405 ± 0.203 | 0.528 ± 0.328 | -0.274  (-0.443 – -0.117) | 0.042  (-0.352 – 0.350) |  | Simple |  |
|  | CPB_X09A | 16.1889 | -61.6004 | 39 | 2.167 ± 0.618 | 0.458 ± 0.200 | 0.562 ± 0.324 | -0.229  (-0.417 – -0.020) | -0.033  (-0.285 – 0.237) |  | Simple |  |
|  | CPB_X24A | 16.1891 | -61.5990 | 36 | 2.000 ± 0.970 | 0.341 ± 0.290 | 0.438 ± 0.386 | -0.299  (-0.486 – -0.126) | 0.284  (-0.011 – 0.583) |  | Extended |  |
|  | CPB_Y08A | 16.1893 | -61.6010 | 45 | 2.500 ± 0.707 | 0.478 ± 0.170 | 0.590 ± 0.260 | -0.228  (-0.361 – -0.108) | -0.073  (-0.294 – 0.132) |  | Extended |  |
|  | CPB_Y13A | 16.1895 | -61.6006 | 39 | 2.167 ± 0.618 | 0.468 ± 0.187 | 0.639 ± 0.326 | -0.361  (-0.545 – -0.168) | -0.133  (-0.392 – 0.135) |  | Extended |  |
| **Petit-Bourg  Forest Site  (CPB)** | FPB_X16A | 16.1984 | -61.6710 | 50 | 2.778 ± 0.647 | 0.548 ± 0.154 | 0.653 ± 0.276 | -0.170  (-0.352 ± 0.042) | -0.185  (-0.443 – 0.084) |  | Extended |  |
|  | FPB_X19D | 16.1982 | -61.6706 | 46 | 2.556 ± 0.705 | 0.500 ± 0.195 | 0.639 ± 0.353 | -0.243  (-0.438 – -0.049) | -0.088  (-0.339 – 0.162) |  | Extended |  |
|  | FPB_Y10B | 16.1983 | -61.6714 | 39 | 2.167 ± 0.924 | 0.415 ± 0.254 | 0.583 ± 0.371 | -0.429  (-0.605 – -0.246) | 0.053  (-0.196 – 0.315) |  | Extended |  |
|  | FPB_Y16A | 16.1980 | -61.6710 | 38 | 2.111 ± 0.832 | 0.375 ± 0.256 | 0.528 ± 0.403 | -0.395  (-0.603 – -0.189) | 0.115  (-0.202 - 0.419) |  | Extended |  |
|  | FPB_Y25A | 16.1977 | -61.6702 | 48 | 2.667 ± 0.767 | 0.480 ± 0.192 | 0.618 ± 0.311 | -0.247  (-0.357 – -0.136) | -0.095  (-0.308 – 0.122) |  | Extended |  |
| **Baie-Mahault Urban Site (CBM)** | CBM_X12A | 16.2478 | -61.5844 | 35 | 1.944 ± 0.725 | 0.356 ± 0.251 | 0.507 ± 0.403 | -0.401  (-0.628 – -0.171) | 0.108  (-0.261 – 0.46) |  | Extended |  |
|  | CBM_X22A | 16.2471 | -61.5838 | 46 | 2.556 ± 0.616 | 0.501 ± 0.147 | 0.569 ± 0.228 | -0.137  (-0.269 – 0.023) | -0.014  (-0.173 – 0.159) |  | Simple |  |
|  | CBM_Y13A | 16.2480 | -61.5838 | 43 | 2.389 ± 0.778 | 0.490 ± 0.209 | 0.611 ± 0.312 | -0.245  (-0.407 – -0.093) | -0.152  (-0.474 – 0.177) |  | Simple |  |
|  | CBM_Y17A | 16.2477 | -61.5838 | 41 | 2.278 ± 0.752 | 0.437 ± 0.229 | 0.521 ± 0.336 | -0.166  (-0.324 – 0.001) | 0.112  (-0.121 – 0.388) |  | Extended |  |
|  | CBM_Y25A | 16.2472 | -61.5833 | 43 | 2.389 ± 0.608 | 0.482 ± 0.139 | 0.562 ± 0.364 | -0.063  (-0.330 – 0.224) | -0.112  (-0.565 – 0.277) |  | Extended |  |
| **Baie-Mahault Forest Site (FBM)** | FBM_X01A | 16.2238 | -61.6829 | 41 | 2.278 ± 0.669 | 0.434 ± 0.210 | 0.569 ± 0.338 | -0.282  (-0.451 – -0.113) | -0.071  (-0.450 – 0.243) |  | Extended |  |
|  | FBM_X08A | 16.2235 | -61.6833 | 43 | 2.389 ± 0.608 | 0.501 ± 0.106 | 0.674 ± 0.239 | -0.335  (-0.480 – -0.174) | -0.247  (-0.475 – -0.015) |  | Simple |  |
|  | FBM_X15B | 16.2232 | -61.6840 | 46 | 2.556 ± 0.922 | 0.472 ± 0.197 | 0.625 ± 0.309 | -0.324  (-0.501 – -0.152) | -0.143  (-0.545 – 0.185) |  | Extended |  |
|  | FBM_X22B | 16.2229 | -61.6844 | 43 | 2.389 ± 0.850 | 0.484 ± 0.234 | 0.597 ± 0.365 | -0.244  (-0.463 – -0.0013) | -0.075  (-0.389 – 0.253) |  | Extended |  |
|  | FBM_Y01B | 16.2243 | -61.6831 | 38 | 2.111 ± 0.758 | 0.389 ± 0.252 | 0.493 ± 0.360 | -0.259  (-0.447 – -0.075) | 0.105  (-0.265 – 0.442) |  | Simple |  |
| **Total** | ***CSR (n = 5, N = 41)*** | | | 67 | 2.541 ± 0.273 | 0.493 ± 0.033 | 0.584 ± 0.076 | -0.183  (-0.279 – -0.08) | -0.012  (-0.116 – 0.099) | 0.148  (0.099 – 0.198) |  |  |
|  | ***FSR (n = 25, N = 213)*** | | | 76 | 1.989 ± 0.162 | 0.351 ±0.047 | 0.424 ± 0.071 | -0.194  (-0.239 – -0.147) | 0.069  (0.006 – 0.123) | 0.226  (0.194 – 0.260) |  |  |
|  | ***CPB (n = 5, N = 40)*** | | | 59 | 2.200 ± 0.183 | 0.429 ± 0.057 | 0.551 ± 0.075 | -0.273  (-0.356 – -0.189) | -0.005  (-0.096 – 0.093) | 0.212  (0.159 – 0.264) |  |  |
|  | ***FPB (n = 5, N = 40)*** | | | 64 | 2.456 ± 0.300 | 0.464 ± 0.069 | 0.604 ± 0.051 | -0.255  (-0.370 – -0.125) | -0.094  (-0.215 – 0.026) | 0.143  (0.109 – 0.178) |  |  |
|  | ***CBM (n = 5, N = 40)*** | | | 62 | 2.311 ± 0.228 | 0.453 ± 0.059 | 0.554 ± 0.042 | -0.181  (-0.284 – -0.068) | 0.044  (-0.064 – 0.150) | 0.203  (0.161 – 0.256) |  |  |
|  | ***FBM (n = 5, N = 40)*** | | | 61 | 2.344 ± 0.164 | 0.456 ± 0.044 | 0.592 ± 0.067 | -0,305  (-0.406 – -0.204) | -0.030  (-0.142 – 0.082) | 0.215  (0.162 – 0.275) |  |  |
|  | ***Urban (n = 15, N = 121)*** | | | 79 | 2.351 ± 0.259 | 0.459 ± 0.055 | 0.563 ± 0.063 | -0.208  (-0.271 – -0.143) | 0.079  (0.006 – 0.150) | 0.243  (0.213 – 0.275) |  |  |
|  | ***Forest (n = 35, N = 293)*** | | | 86 | 2.107 ± 0.262 | 0.382 ± 0.069 | 0.473 ± 0.104 | -0.220  (-0.266 – -0.170) | 0.076  (0.004 – 0.140) | 0.255  (0.224 – 0.292) |  |  |

**Table S4.** Results of linear mixed-effects models (LMM) testing the effect of habitat type (urban vs forest) on genetic diversity indices. Results are shown for the global model and for each pair separately. *Ar* = allelic richness; *He* = expected heterozygosity; *Ho* = observed heterozygosity; *F_IC_* = intra-nest inbreeding coefficient. Global model: estimate and SE of the habitat fixed effect; *χ²* and *p* from likelihood ratio tests (LRT, df = 1). Pair-specific model: estimates and SE from emmeans contrasts (df = 44 for all comparisons); *p* (Bonferroni) = *p*-values corrected across simultaneous comparisons. Bold values indicate *p* < 0.05**.**

| **Index** | **Global model** | | | |  | **Pair-specific model** | | | | |  |
| --- | --- | --- | --- | --- | --- | --- | --- | --- | --- | --- | --- |
|  | **Estimate (Urban-Forest)** | **SE** | ***χ²*** | ***p*** | **Pair** | **Estimate (Urban-Forest)** | **SE** | **t** | ***p*** | ***p* (Bonferroni)** |  |
| *Ar* | +0.098 | 0.152 | 0.399 | 0.528 | SR | +0.552 | 0.097 | 5.663 | **< 0.001** | **< 0.001** |  |
|  |  |  |  |  | PB | -0.256 | 0.126 | -2.032 | **0.048** | 0.145 |  |
|  |  |  |  |  | BM | -0.033 | 0.126 | -0.265 | 0.792 | 0.920 |  |
| *He* | +0.039 | 0.037 | 0.995 | 0.319 | SR | +0.142 | 0.025 | 5.777 | **< 0.001** | **< 0.001** |  |
|  |  |  |  |  | PB | -0.034 | 0.032 | -1.063 | 0.294 | 0.504 |  |
|  |  |  |  |  | BM | -0.003 | 0.032 | -0.094 | 0.926 | 0.920 |  |
| *Ho* | +0.031 | 0.055 | 0.294 | 0.588 | SR | +0.160 | 0.033 | 4.849 | **< 0.001** | **< 0.001** |  |
|  |  |  |  |  | PB | -0.053 | 0.043 | -1.235 | 0.223 | 0.504 |  |
|  |  |  |  |  | BM | -0.037 | 0.043 | -0.878 | 0.385 | 0.577 |  |
| *F_IC_* | +0.025 | 0.030 | 0.679 | 0.410 | SR | +0.012 | 0.059 | 0.199 | 0.843 | 0.920 |  |
|  |  |  |  |  | PB | +0.019 | 0.076 | 0.245 | 0.808 | 0.920 |  |
|  |  |  |  |  | BM | +0.086 | 0.076 | 1.140 | 0.246 | 0.504 |  |

**Table S5.** Results of the global MLPE model testing the effects of geographic distance and habitat comparison on pairwise genetic differentiation (*F_CT_*).

| **Predictors** | **Estimate** | **SE** | **t** | **df** | ***p*** |  |
| --- | --- | --- | --- | --- | --- | --- |
| Intercept | 0.296 | 0.024 | 12.44 | 265 | <0.001 |  |
| log(dist) | 0.001 | 0.009 | 0.15 | 511 | 0.878 |  |
| Inter-habitat comparison | 0.128 | 0.042 | 3.08 | 335 | **0.002** |  |

**References for the Appendix A**

Amatulli, G., Domisch, S., Tuanmu, M.-N., Parmentier, B., Ranipeta, A., Malczyk, J., & Jetz, W. (2018). A suite of global, cross-scale topographic variables for environmental and biodiversity modeling. *Scientific Data*, *5*(1), 180040. https://doi.org/10.1038/sdata.2018.40

Lembrechts, J. J., van den Hoogen, J., Aalto, J., Ashcroft, M. B., De Frenne, P., Kemppinen, J., Kopecký, M., Luoto, M., Maclean, I. M. D., Crowther, T. W., Bailey, J. J., Haesen, S., Klinges, D. H., Niittynen, P., Scheffers, B. R., Van Meerbeek, K., Aartsma, P., Abdalaze, O., Abedi, M., … Lenoir, J. (2022). Global maps of soil temperature. *Global Change Biology*, *28*(9), 3110–3144. https://doi.org/10.1111/gcb.16060

Liu, R., & Liu, Y. (2024). *GLOBMAP FTC: A global annual fractional tree cover dataset since 2000* [Dataset]. Zenodo. https://zenodo.org/records/10589730

Elvidge, C. D., Zhizhin, M., Ghosh, T., Hsu, F.-C., & Taneja, J. (2021). Annual time series of global VIIRS nighttime lights derived from monthly averages: 2012 to 2019. *Remote Sensing*, *13*(5), 922. https://doi.org/10.3390/rs13050922

Wang, X., Meng, X., & Long, Y. (2022). Projecting 1 km-grid population distributions from 2020 to 2100 globally under shared socioeconomic pathways. *Scientific Data*, *9*(1), 563. https://doi.org/10.1038/s41597-022-01675-x
